# Cellular N-Myristoyl Transferases Are Required for Mammarenavirus Multiplication

**DOI:** 10.3390/v16091362

**Published:** 2024-08-26

**Authors:** Haydar Witwit, Carlos Alberto Betancourt, Beatrice Cubitt, Roaa Khafaji, Heinrich Kowalski, Nathaniel Jackson, Chengjin Ye, Luis Martinez-Sobrido, Juan C. de la Torre

**Affiliations:** 1 Department of Immunology and Microbiology, The Scripps Research Institute, La Jolla, CA 92037, USA; hwitwit@scripps.edu (H.W.); 9218carlos@gmail.com (C.A.B.); bcubitt@scripps.edu (B.C.); rkhafaji@scripps.edu (R.K.); 2 Center for Medical Biochemistry, Max F. Perutz Laboratories (MFPL), Medical University of Vienna, Vienna Biocenter (VBC), 1030 Vienna, Austria; heinrich.kowalski@meduniwien.ac.at; 3 Texas Biomedical Research Institute, San Antonio, TX 78227, USA; njackson@txbiomed.org (N.J.); lmartinez@txbiomed.org (L.M.-S.)

**Keywords:** mammarenavirus, myristoylation, N-myristoyltransferases, antiviral, LCMV, LASV, proteasome

## Abstract

The mammarenavirus matrix Z protein plays critical roles in virus assembly and cell egress. Meanwhile, heterotrimer complexes of a stable signal peptide (SSP) together with glycoprotein subunits GP1 and GP2, generated via co-and post-translational processing of the surface glycoprotein precursor GPC, form the spikes that decorate the virion surface and mediate virus cell entry via receptor-mediated endocytosis. The Z protein and the SSP undergo N-terminal myristoylation by host cell N-myristoyltransferases (NMT1 and NMT2), and G2A mutations that prevent myristoylation of Z or SSP have been shown to affect the Z-mediated virus budding and GP2-mediated fusion activity that is required to complete the virus cell entry process. In the present work, we present evidence that the validated on-target specific pan-NMT inhibitor DDD85646 exerts a potent antiviral activity against the prototypic mammarenavirus lymphocytic choriomeningitis virus (LCMV) that correlates with reduced Z budding activity and GP2-mediated fusion activity as well as with proteasome-mediated degradation of the Z protein. The potent anti-mammarenaviral activity of DDD85646 was also observed with the hemorrhagic-fever-causing Junin (JUNV) and Lassa (LASV) mammarenaviruses. Our results support the exploration of NMT inhibition as a broad-spectrum antiviral against human pathogenic mammarenaviruses.

## 1. Introduction

Mammarenaviruses are enveloped viruses with a bi-segmented negative-stranded (NS) RNA genome [1]. Each of the two genome RNA segments, L (ca 7.3 kb) and S (ca 3.5 kb), uses an ambisense coding strategy to direct the synthesis of two polypeptides in opposite orientations, separated by a non-coding intergenic region (IGR). The L genome segment encodes the viral RNA-dependent RNA polymerase (L) and the Z matrix protein, whereas the S genome segment encodes the viral glycoprotein precursor (GPC) and the viral nucleoprotein (NP). GPC is co-translationally cleaved by a signal peptidase to produce a 58-amino acid stable signal peptide (SSP) and a GP that is post-translationally processed by the cellular site 1 protease (S1P) to yield the mature GP1 and GP2 subunits that, together with the SSP, form the spikes that decorate the virus surface and mediate cell entry via receptor-mediated endocytosis [2,3,4,5]. GP1 mediates binding to the cellular receptor and GP2 mediates the pH-dependent fusion event in the late endosome that is required for the release of virus ribonucleoprotein (vRNP) complexes into the cell cytoplasm, where they direct the replication and transcription of the viral genome [6,7].

Early studies showed that myristic acid analogs inhibited multiplication of the mammarenavirus Junin (JUNV) [8]. Subsequently, N-myristoylation was shown to be required for the role of the mammarenavirus matrix Z protein in assembly and budding [9] and for the role of the SSP in the GP2-mediated fusion event [10]. These findings were based on the use of 2-hydroxy-myristic acid (2-HMA) and 2-HMA analogs as inhibitors of the N-myristoyltransferases (NMT1 and NMT2) responsible for catalyzing N-myristoylation in mammalian cells [8,9]. However, recent studies have demonstrated that 2-HMA acts off-target and does not inhibit N-myristoylation in a concentration range consistent with its activity toward NMT [11]. Therefore, we have revisited the contribution of N-myristoylation in mammarenavirus infection using the validated on-target specific NMT1/2 inhibitor DDD85646 [12,13]. Here, we present evidence that DDD85646 exhibits very potent antiviral activity against the prototypic mammarenavirus lymphocytic choriomeningitis virus (LCMV) in cultured cells. Cell-based assays probing different steps of the LCMV life cycle indicated that DDD85646 interfered with Z budding activity and the GP2-mediated fusion between viral and cellular membranes, a process that requires the participation of myristoylated SSP [10,14,15]. Consistent with the proposed role of a glycine-specific N-degron pathway in the quality control of protein N-myristoylation [16], treatment with DDD85646 resulted in the targeting of Z for degradation via the proteasome pathway, which may have also contributed to the robust DDD85646-mediated inhibition of the production of infectious virus progeny.

The potent anti-LCMV activity of DDD85646 was also observed with other mammarenaviruses, including Lassa virus (LASV) and Junin virus (JUNV), the causative agents of Lassa fever (LF) and Argentine hemorrhagic fever (AHF) diseases that pose important public health problems in their endemic regions [17,18,19,20,21,22]. This suggests that inhibition of NMT can possibly be exploited as a broad-spectrum antiviral agent against human pathogenic mammarenaviruses. It should be noted that LASV is highly prevalent in Western Africa, where it is estimated to infect several hundred thousand people yearly, resulting in a high number of LF cases associated with high morbidity and mortality. To date, there are no FDA-approved mammarenavirus-specific therapeutics, and current LASV therapy is limited to the off-label use of ribavirin, for which efficacy remains controversial [23]. Several small molecules, including the broad-spectrum mammarenavirus RNA-directed RNA polymerase inhibitor favipiravir and the mammarenavirus glycoprotein GP2-mediated fusion inhibitor ST-193, have shown promising anti-mammarenaviral effects in various animal models of mammarenavirus-induced human diseases [24,25,26,27]. Nevertheless, the identification and characterization of additional safe and effective anti-mammarenaviral drugs can facilitate the implementation of combination therapy, an approach known to counteract the emergence of drug-resistant variants often observed with monotherapy strategies. Moreover, the emergence of viral variants resistant to host-targeting antivirals (HTAs) is usually significantly reduced or entirely absent, but HTAs can be associated with significant side effects. However, side effects associated with the use of HTAs might be manageable in the case of acute infections, such as HF disease caused by mammarenaviruses, where the duration of the treatment would be rather short.

Aberrant NMT expression has been identified in cancer cells, and inhibition of myristoylation is being actively pursued as a novel anticancer treatment strategy. Accordingly, the small molecule NMT inhibitor PCLX-001 has been shown to be safe and well tolerated in humans [28,29], supporting the interest in exploring the repurposing of NMT inhibitors to treat infections by human pathogenic mammarenaviruses.

## 2. Materials and Methods

### 2.1. Cells and Viruses

*Grivet* (*Chlorocebus aethiops*) Vero E6 (ATCC CRL-1586), Homo sapiens A549 (ATCC CCL-185), and HEK 293T (ATCC CRL-3216) cell lines were maintained in Dulbecco’s modified Eagle medium (DMEM) (ThermoFisher Scientific, Waltham, MA, USA) containing 10% heat-inactivated fetal bovine serum (FBS), 2 mM L-glutamine, 100 μg/mL streptomycin, and 100 U/mL penicillin. HAP1 cells were obtained from Horizon Discovery and grown in IMDM supplemented with 10% FBS and 1% Pen-Strep. CRISPR/Cas9-mediated NMT1 and NMT2 knockout (KO) HAP1 cell lines have been described [12]. The recombinant LCMV expressing the green fluorescent protein (GFP) fused to the LCMV NP via a P2A ribosomal skipping sequence (rLCMV/GFP-P2A-NP, referred to as rLCMV/GFP); a single-cycle infectious rLCMV expressing the *Zoanthus* sp. green fluorescent protein (ZsG) fused to the LCMV NP via a P2A ribosomal skipping sequence (rLCMV∆GPC/ZsG-P2A-NP, here referred to as rLCMV∆GPC/ZsG) [30]; the tri-segmented form of the live attenuated vaccine strain Candid#1 of JUNV expressing the green fluorescent protein (GFP, r3Can/GFP) [31]; the tri-segmented form of TCRV expressing GFP (r3TCRV/GFP) [32]; the reassortant ML29 expressing GFP (r3ML29/GFP) [33]; and the tri-segmented form of the LASV Josiah strain [34] have been described. The rLCMV/Z-HA was generated using described procedures [35], but with the L segment containing a C-terminal HA-tagged version of Z.

### 2.2. Compounds

DDD85646 (2,6-Dichloro-4-[2-(1-piperazinyl)-4-pyridinyl]-N-(1,3,5-trimethyl-1H-pyrazol-4-yl)-benzenesulfonamide, CAS No. 1215010-55-1) was synthesized as described [12,13], dissolved in DMSO at 25 mM, and kept in aliquots at –20 °C. IMP-1088 (5-[3,4-difluoro-2-[2-(1,3,5-trimethyl-1H-pyrazol-4-yl)ethoxy]phenyl]-N,N,1-trimethyl-1H-indazole-3-methanamine, Cat No. 25366-1) was purchased from Cayman Chemical, dissolved in methyl acetate at 11 mM, and kept in aliquots at −20. Clemizole hydrochloride (MFG No. HY-30234A-5MG, MedChemExpress, NJ, USA) was purchased from Fisher Scientific (Cat. No. 502082225). F3406-2010 was purchased from DC Chemicals (Cat. No. DC11877). Ribavirin (Cat. No. R9644) and MG132 (MFG No. M7449) were purchased from Sigma-Aldrich (St. Louis, MO, USA). Sodium citrate buffer (0.1M, pH 5.0, Sterile) was purchased from bioWorld (MFG No. 40121003-1, Dublin, OH, USA). FluoroBrite DMEM was purchased from Life Technologies (Cat. No. A1896701, Carlsbad, CA, USA).

### 2.3. Cell Cytotoxicity Assay and CC_50_ Determination

Cell viability was assessed using the CellTiter 96 AQueous One Solution Reagent (Promega, Madison, WI, USA). This method determines the number of viable cells based on the conversion of a formazan product from 3-(4,5-dimethylthiazol-2-yl)-5-(3-carboxymethoxyphenyl)-2-(4-sulfophenyl)-2H-tetrazolim by nicotinamide adenine dinucleotide phosphate (NADPH) or nicotinamide adenine dinucleotide phosphate (NADH) generated in living cells. A549 cells were plated on 96-well clear-bottom plates (4.0 × 10^4^ cells/well). Serial dilutions (2-fold) of each compound were added to the cells, and at 72 h after the drug treatment, CellTiter 96 AQueous One Solution Reagent was added and incubated for 35 min (37 °C and 5% CO_2_). Signals were quantified using the Cytation 5 reader (BioTek, Winooski, VT, USA). The resulting optical densities were normalized to the vehicle-treated (DMSO) control samples, which were assigned a value of 100%. The half-maximal cytotoxic concentrations (CC_50_) were determined using GraphPad Prism, v10 (Prism10).

### 2.4. EC_50_ Determination

For the EC_50_ determination, cells were seeded on 96-well clear-bottom black plates (4.0 × 10^4^ cells/well) and infected (MOI 0.05) 20 h later with rLCMV/GFP-P2A-NP. After a 90 min adsorption, the virus inoculum was aspirated off and compound-containing media was added to the cells. At 72 h pi, the cells were fixed with 4% paraformaldehyde, and GFP expression was determined by fluorescence using a fluorescent plate reader (Cytation 5, BioTek, Winooski, VT, USA). The mean relative fluorescence units were normalized to vehicle control (DMSO)-treated cells, which were assigned a value of 100%. The half-maximal effective concentrations (EC_50_) were determined using Prism10. The selectivity index (SI) for each compound was determined using the ratio CC_50_/EC_50_.

### 2.5. Viral Growth Kinetics

For growth kinetics, virus was added to cells (500 µL/well in M12-well plates) at the indicated MOI. After 90 min of adsorption (37 °C and 5% CO_2_), the virus inocula were removed, the cells were washed once with DMEM 2% FBS before fresh media containing the indicated compounds and concentrations was added, and the infection was allowed to proceed at 37 °C and 5% CO_2_. At the indicated times post-infection (pi), cell culture supernatants (CCS) were collected, and viral titers were determined using a focus-forming assay (FFA) [36].

### 2.6. LCMV Minigenome Assay

The LCMV minigenome (MG) assay was performed as described [37]. Briefly, HEK 293T cells were cultured on poly-L-lysine-treated M12-well plates (4.5 × 10^5^ cells/well). Cells were transfected using Lipofectamine 2000 (2.5 µL/µg of DNA) (ThermoFisher Scientific) with Pol II-based expression plasmids (pCAGGS) for T7 RNA polymerase (pC-T7, 0.5 µg), NP (pC-NP, 0.3 µg), and L (pC-L, 0.3 µg) together with a plasmid directing the intracellular synthesis of an LCMV MG expressing the chloramphenicol acetyl transferase (CAT) reporter gene under a T7 promoter (pT7-MG/CAT, 0.5 µg). After 5 h, the transfection mixture was replaced with fresh media and incubated for 72 h at 37 °C and 5% CO_2_. At 72 h post-transfection, whole-cell lysates were harvested to determine the protein expression levels of CAT using a CAT ELISA kit (Roche, Sydney, Australia). Briefly, whole-cell lysates were prepared with 0.5 mL of lysis buffer, and 10 µL of each sample was used for the reaction. Diluted samples were added to the CAT ELISA plates and incubated for 1 h at 37 °C. After incubation with the samples, the plates were washed, and the primary antibody (anti-CAT-digoxigenin) and secondary antibody (anti-CAT-digoxigenin-peroxidase) were added sequentially followed by the substrate. After 20 min, absorbance was measured using the ELISA reader at 405 nm for the tested samples and at 490 nm for the reference sample.

### 2.7. Budding Assay

The luciferase-based budding assay was performed as described [38]. Briefly, HEK 293T cells were seeded on poly-L-lysine-coated M12-well plates (3.5 × 10^5^ cells/well). After overnight incubation, 2 µg of DNA of either pC-LCMV-Z-Gaussia luciferase (GLuc), pC-LCMV-mutant Z [G2A]-GLuc, or pC-LASV-Z was transfected using Lipofectamine 2000 (2.5 µL/µg of DNA). After 5 h, the transfection mixtures were replaced with fresh media containing the indicated compounds. After 48 h, cell culture supernatants (CCSs) containing virion-like particles (VLPs) were harvested and clarified by low-speed centrifugation to remove cell debris. Aliquots (20 µL each) from the CCS samples were added to 96-well black plates (VWR, West Chester, PA, USA), and 50 µL of the SteadyGlo luciferase reagent (Promega) was added to each well. Whole-cell lysates (WCL) from the same samples were processed to determine the cell-associated activity of GLuc. Luminescence signals were measured using the Berthold Centro LB 960 luminometer (Berthold Technologies, Oak Ridge, TN, USA). The activity (relative light units) of GLuc in the CCS and WCL was used as a surrogate for Z expression. Budding efficiency was defined as the ratio Z_VLP_/Z_VLP_ + Z_WCL_.

### 2.8. Western Blotting

Whole-cell lysates were prepared in lysis buffer (250 mM NaCl, 50 mM Tris-HCl (pH 7.5), 0.5% Triton X-100, 10% glycerol). Samples were denatured for 5 min at 95 °C. Then, 12.5 μg of each sample was separated by SDS-PAGE using a stain-free gel (Bio-Rad, Hercules, CA, USA), transferred to a low-fluorescence PVDF membrane (Bio-Rad, Hercules, CA, USA), and immunoblotted with anti-HA (Genscript, Piscataway, NJ, USA). Bands were visualized with a chemiluminescent substrate (ThermoFisher Scientific).

### 2.9. Virus Titration

Virus titers were determined by FFA [36]. Serial (10-fold) dilutions of the samples were performed in DMEM containing 2% FBS and used to infect Vero E6 cell monolayers in 96-well plates (2 × 10^4^ cells/well). At 20 h pi, the cells were fixed with 4% paraformaldehyde in phosphate-buffered saline. Foci of cells infected with rLCMV/GFP were determined by epifluorescence.

### 2.10. RNA Isolation and Characterization

Total cellular RNA was isolated using the TRI reagent (TR 118) (Molecular Research Center, Cincinnati, OH, USA) according to the manufacturer’s instructions, resuspended in sodium citrate pH 6.4, and stored at −80 °C. RNA samples were analyzed by Northern blot hybridization or RT-qPCR.

**Northern blotting:** RNA samples (4 μg) were fractionated by 2.2 M formaldehyde–agarose (1.2%) gel electrophoresis. The gel was washed once each with warm H_2_O and 10 mM NaPO_4_, and the RNA was transferred in 20X SSC (3 M sodium chloride, 0.3 M sodium citrate) to a Magnagraph membrane (NJTHYA0010, Osmonics MagnaGraph nylon) using the rapid downward transfer system (TurboBlotter). Membrane-bound RNA was cross-linked by exposure to UV light (automated 12 s duration, twice), and then the membrane was washed with MilliQ water and stained with methylene blue (MB) to reveal the 18S and 28S RNA plus RNA ladder. After image acquisition by Image Quant LAS 4000 (GE Healthcare), a 1% SDS solution was used to remove the MB staining, and the membrane was hybridized using QuickHyb (Cat. No. 201220–12, Agilent, Santa Clara, CA, USA) to a ^32^P-labeled dsDNA NP. Hybridization was performed at 65 °C overnight. The DNA probe was prepared according to the supplier’s protocol using a DecaPrime kit (Cat. No. AM1455, Ambion, Life Technologies, Carlsbad, USA). After overnight hybridization, the membrane was washed twice with 2X SSC–1% SDS at 65 °C, followed by two washes with 0.2X SSC–0.1% SDS at 65 °C, and then exposed to X-ray film using Typhoon Trio Imager (GE Healthcare).

**RT-qPCR:** RNA (1 μg) was reverse-transcribed to cDNA using the SuperScript™ IV first-strand synthesis system (Thermo Fisher Scientific). To amplify LCMV NP and the housekeeping gene GAPDH, Powerup SYBR (A25742, Life Technologies) was used. The following primers were used for the amplification: NP forward (F): 5′ CAGAAATGTTGATGCTGGACTGC-3′ and NP reverse (R): 5′-CAGACCTTGGCTTGCTTTACACAG-3′ [39]; GAPDH F: 5′-CATGAGAAGTATGACAACAGCC-3′ and GAPDH R: 5′-TGAGTCCTTCCACGATACC-3′; ISG15 F: 5′-CAGGACGACCTGTTCTGGC-3′ and ISG15 R: 5′-GATTCATGAACACGGTGCTCAGG-3′; and MX1 F: 5′-GCAGCTTCAGAAGGCCATGC-3′ and MX1 R: 5′-CCTTCAGGAACTTCCGCTTGTC-3′.

### 2.11. GP2-Mediated Fusion Assay

HEK293T cells were plated on poly-L-lysine-treated 24-well plates (2.5 × 10^5^ cells/well). The next day, the cells were transfected with a pCAGGS plasmid expressing GFP protein (50 ng/well), along with either an empty pCAGGS plasmid or pCAGGS plasmids expressing LCMV GPC or LASV GPC proteins (1 µg/well). Lipofectamine 3000 reagent was used for the transfection according to the manufacturer’s protocol. Cells were incubated with the transfection mixture for 5 h, washed once with media, and then replaced with media with or without DDD85646 (5 µM). At 24 h post-transfection, the cells were treated with acidified (pH 5) or neutral (pH 7.2) media for 15 min followed by a wash with DMEM, and then they were returned to DMEM containing 10% FBS and monitored over time for the appearance of syncytia using a fluorescence microscope. Cells were fixed with 4% PFA, washed with DPBS, and imaged at 20× magnification using a Keyence BZ-X710 microscope.

### 2.12. Assessment of NP to Z Ratio

A549 cells were seeded onto 96-well clear bottom plates (4.0 × 10^4^ cells/well), infected with rLCMV/Z-HA (MOI 1), and treated with serial dilutions (3-fold) of the indicated compound. At 72 h after drug treatment, the cells were fixed with 4% PFA. NP was stained with the Alexa Fluor 488-conjugated VL4 rat monoclonal antibody against NP (Bio C Cell, West Lebanon, NH, USA) and the Z protein was stained with an anti-HA antibody (Cat No. A01244, Genscript, Piscataway NJ, USA) conjugated to Alexa Fluor 647. DNA was probed with DAPI. The resulting optical signals were normalized to the vehicle (DMSO) control group, which was adjusted to 100%. Results were plotted using Prism10.

### 2.13. Immunofluorescence and Subcellular Localization of NP and Z

A549 cells were plated on 96-well clear bottom optical plates (4.0 × 10^4^ cells/well). The next day, the cells were infected with rLCMV/Z-HA (MOI 0.05) for 90 min before the media was aspirated and replaced with DDD85646 (5 μM)-containing media, and then at 72 h after drug treatment, the cells were fixed with 4% PFA. NP was identified with the rat monoclonal antibody VL4 (Bio C Cell, West Lebanon, NH, USA) conjugated to Alexa Fluor 488, and the Z protein was stained with an anti-HA antibody (Cat No. A01244, Genescript, Princeton, NJ, USA) conjugated to Alexa Fluor 568. DNA was probed with DAPI. HEK293T cells were plated on poly-L-Lysine 96-well clear-bottom optically treated plates (2.0 × 10^4^ cells/well). The next day, the cells were transfected with LCMV Z-HA or Z-G2A-HA plasmids, and at 24 h post-transfection (h pt), the media was aspirated and replaced with DDD85646 (5 μM)-containing media. At 48 h after drug treatment, the cells were fixed with 4% PFA. Z protein was stained with an anti-HA antibody (Cat No. A01244, Genescript, Princeton, NJ, USA) conjugated to Alexa Fluor 568, and DNA was probed with DAPI.

### 2.14. Statistical Analyses

All statistical analyses were conducted, as indicated in the respective assays, using GraphPad Prism software v10 (GraphPad Software, Boston, MA, USA, www.graphpad.com).

## 3. Results

### 3.1. Dose-Dependent Effect of the NMT1/2 Specific Inhibitor DDD85646 on LCMV Multiplication

**Figure 1:**
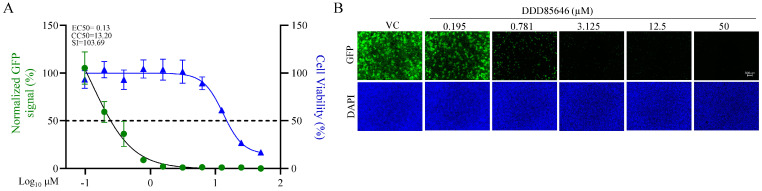
**Dose-dependent effect of the NMT inhibitor DDD85646 on LCMV multiplication in A549 cells**. A549 cells were seeded at 4 × 10^4^ cells/well into a 96-well plate, infected with rLCMV/WT-GFP-P2A-NP (MOI 0.05), and treated with DDD85646 at the indicated concentrations. At 72 h pi, the cells were fixed, and the number of infected cells was determined by immunofluorescence. Numbers of infected cells were normalized to those of vehicle control (VC)-infected cells and expressed as a % of infected cells (**A**). The total viable cell number was determined by an MTS/formazan bioreduction assay using the Cytation 5 reader. Results show the percentage of infected cells (four biological replicates). EC_50_ and CC_50_ values were calculated using a variable slope (based on four parameters) model (Prism10). Results show the mean and SD of four biological replicates. Representative immunofluorescence images of the dose-response assay of selected doses of DDD85646 are shown (**B**). DAPI was used to stain the DNA (nuclei). Images were taken at 4× magnification using a Keyence BZ-X710 fluorescence microscope.

DDD85646, a validated potent and selective pan NMT inhibitor [13,40], exhibited a potent dose-dependent inhibitory effect on rLCMV/GFP-P2A-NP multiplication in A549 cells, with an EC_50_ value of 0.13 μM (effective drug concentration that reduced GFP expression to 50% when compared to that of vehicle-treated controls) (Figure 1). The inhibitory effect of DDD85646 on LCMV multiplication was not a consequence of drug-induced cell toxicity, as the CC_50_ (drug concentration that reduced cell viability by 50% when compared with that of vehicle-treated cells) of DDD85646 was >13 μM (Figure 1), resulting in a DDD85646 selectivity index (SI = CC_50_/EC_50_) of >100.

### 3.2. Effect of DDD85646 on LCMV Multi-Step Growth Kinetics and Peak Titers

**Figure 2:**
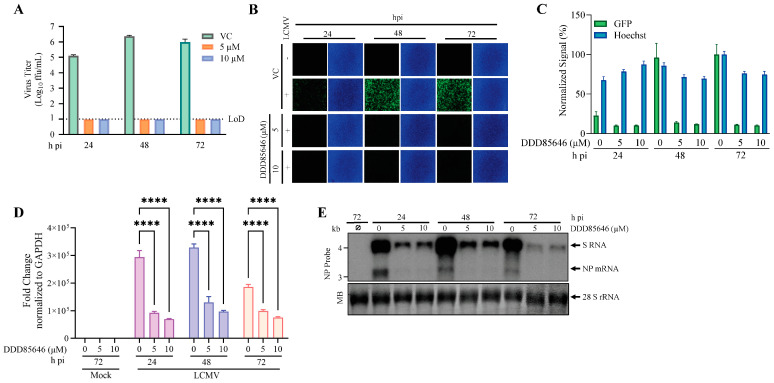
**Effect of the NMT inhibitor DDD85646 on LCMV multi-step growth kinetics and peak titers in A549 cells.** (**A**) Effect of DDD85646 on the production of infectious viral progeny. A549 cells were seeded at 2 × 10^5^ cells/well in an M12-well plate, infected with rLCMV/GFP-P2A-NP (MOI 0.05), and treated with DDD85646 (5 or 10 µM) or with VC. At the indicated time points, cell culture supernatants were collected, and the titers of infectious virus were determined by the focus-forming assay (FFA) using Vero E6 cells. (**B**,**C**). Effect of DDD85646 on virus propagation. At the indicated h pi, samples from A were switched to FluoroBrite DMEM containing Hoechst dye (5 µg/mL) and stained for 15 min for live imaging fluorescence (**B**). GFP signals were determined using Cytation 5 and normalized to the average of the VC (72 h pi). (**C**,**D**) Effect of DDD85646 on viral RNA synthesis. Total cellular RNA was isolated from the samples described in (**B**), and the same RNA amount (10 ng) of each sample was analyzed by RT-qPCR using random hexamers for the RT step, followed by quantitative PCR with specific primers for LCMV NP and the host cell GAPDH. The repeated-measures analysis of variance with mixed-effect analysis and Dunnet’s correction for multiple comparisons were used to determine the statistical significance of the technical triplicates. Statistically significant values: **** *p* < 0.0001. (**E**) Effect of DDD85646 on LCMV replication and transcription. Cells were infected with rLCMV/GFP-P2A-NP (MOI 0.05), and at the indicated h pi, total cellular RNA was isolated and analyzed by Northern blotting using an LCMV NP-specific dsDNA ^32^P probe. Methylene blue (MB) staining was used to confirm similar transfer efficiencies for all the RNA samples.

To examine the effect of DDD85646 on LCMV multi-step growth kinetics in A549 cells, we infected them with rLCMV/GFP-P2A-NP (MOI 0.05) and treated them with the indicated concentrations of DDD85646 or vehicle control (VC). At the indicated hours post-infection (h pi), we collected cell culture supernatant (CCS) samples and determined the virus titers using a focus-forming assay (FFA) (Figure 2A). Treatment with DDD85646 at either 10 or 5 µM resulted in the lack of detection of infectious LCMV progeny, which correlated with restricted LCMV propagation within the cell monolayer in the presence of DDD85646 (Figure 2B,C) and reduced levels of viral RNA synthesis as determined by RT-qPCR (Figure 2D). To assess whether treatment with DDD85646 could differentially affect the levels of LCMV replication and transcription, RNA samples from rLCMV/GFP-P2A-NP-infected (MOI 0.05) cells were analyzed by Northern blotting, using an NP-specific ^32^P-dsDNA probe that detects genome and antigenome S RNA species (replication) and NP mRNA (transcription). Treatment with DDD85646 resulted in reduced levels of virus replication and transcription at all the times examined, starting at 24 h pi (Figure 2E).

### 3.3. Effect of DDD85646 on Different Steps of the LCMV Life Cycle

**Figure 3:**
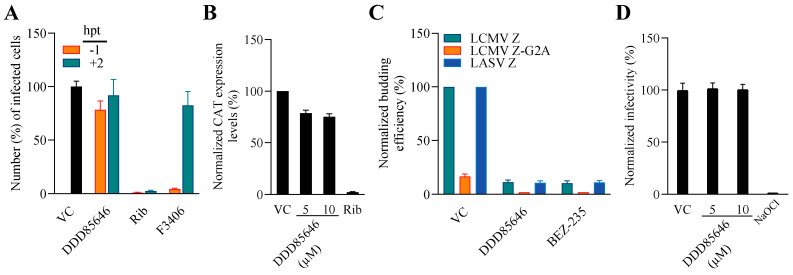
**Effect of DDD85646 on different steps of the LCMV life cycle.** (**A**) Time-of-addition assay: Vero E6 cells were seeded into a 96-well plate at a density of 2 × 10^4^/well. The next day, the cells were infected with the single-cycle infectious rLCMV∆GPC/ZsG (MOI 0.5) and treated with DDD85646 (5 µM) or with VC, starting 1 h before (−1 h) or 2 h after (+2 h) infection. The LCMV cell-entry inhibitor F3406 (10 µM) and ribavirin (Rib) (100 µM) were used as controls. At 48 h pi, ZsG^+^ cells were assessed using the Synergy™ H4 Hybrid Microplate Reader from Biotek. Values were normalized to VC-treated infected cells; the data represent an average of three biological replicates. (**B**) LCMV cell-based MG assay: HEK293T cells were seeded into M24-well plates and transfected with plasmids expressing LCMV MG-CAT together with plasmids expressing the viral trans-acting factors NP, L polymerase, and T7 RNA polymerase and then treated with DDD85646 (5 or 10 µM); Rib- and VC-treated samples were used as controls. At 72 h post-transfection, cell lysates were prepared, and total protein was determined using a BCA protein assay. The same amount of total protein from each sample was used to determine CAT protein expression levels using a CAT ELISA kit (Roche, Sydney, Australia) and normalized by assigning the value of 100% activity to vehicle control-treated samples. (**C**) Z budding activity assay: HEK293T cells were seeded onto poly-L-lysine-coated M12-well plates at 1.75 × 10^5^ cells/ well. The next day, the cells were transfected with either pC-LCMV-Z-GLuc, pC-LASV-Z-Gluc, or pC-LCMV-Z-G2A-GLuc. At 5 h post-transfection, the cells were washed three times and fed with fresh medium containing the relevant drugs at the indicated concentrations. At 48 h post-transfection, cell culture supernatant (CCS) samples were collected, and whole-cell lysates (WCL) were prepared. GLuc activity in the CCS and WCL samples was determined using the SteadyGlo Luciferase Pierce: Gaussia Luciferase Glow assay kit and a Berthold Centro LB 960 luminometer (Berthold Technologies, Oak Ridge, TN, USA). The activity (relative light units) of GLuc in the CCS and WCL was used as a surrogate for Z expression and budding efficiency, defined as the ratio Z_VLP_/Z_VLP_ + Z_WCL_. Budding efficiency values were normalized by assigning the value of 100% to vehicle control-treated samples and plotted using Prism10. (**D**) Virucidal assay: 10^5^ FFU of rLCMV/GFP-P2A-NP was incubated for 30 min at RT in the presence of 0, 5, or 10 µM DDD85646 or in the presence of the validated virucidal compound sodium hypochlorite (0.5%). After treatment, the samples were diluted 1000-fold in DMEM/2%FBS, resulting in concentrations (5 nM and 10 nM) that did not have noticeable anti-LCMV activity, and the number of infectious particles was determined by the FFA.

To gain further insights about the mechanism by which DDD85646 exerted its antiviral activity against LCMV, we examined which steps of the virus life cycle were affected in the presence of DDD85646. To examine whether DDD85646 affected the cell entry or post-entry step of the LCMV life cycle, we conducted a time-of-addition experiment using the single-cycle infectious rLCMV∆GPC/ZsG to prevent the confounding factor introduced by multiple rounds of infection (Figure 3A). Treatment with DDD85646 starting at 1 h prior to virus addition or at 2 h pi did not significantly affect the number of ZsG^+^ cells, whereas treatment with F3406, an inhibitor of LCMV cell entry, starting at –1 h pi, but not at +2 h, resulted in over 90% reduction in the number of ZsG^+^ cells. As expected, treatment with ribavirin (Rib) (100 µM) at –1 h or +2 h pi potently reduced the number of ZsG^+^ cells detected at 48 h pi.

To study the effect of DDD85646 on viral RNA synthesis directed by the LCMV vRNP, we examined the effect of DDD85646 on the activity of a cell-based LCMV minigenome (MG) system (Figure 3B). This MG system recapitulates LCMV RNA synthesis using an intracellularly reconstituted LCMV vRNP expressing the CAT reporter gene. Reconstitution of LCMV vRNP requires co-expression of the LCMV L and NP proteins as well as the LCMV MG vRNA. Expression levels of the MG-directed CAT expression served as a comprehensive measurement of LCMV MG replication, transcription, and translation of the LCMV MG CAT reporter. Compared with vehicle-treated controls, treatment with DDD85646 at 5 or 10 µM had a modest effect on CAT expression, whereas treatment with Rib (100 µM) resulted in very low levels of CAT expression.

The mammarenavirus matrix protein Z has been shown to be the main driving force of virion budding [41]. To assess whether DDD85646 affected the Z-mediated budding process, we used a published cell-based Z budding assay where the activity of the Gaussia luciferase (Gluc) reporter gene serves as a surrogate for Z budding activity [38]. We transfected HEK293T cells with a plasmid expressing LCMV Z-GLuc and treated them with DDD85646 (5 µM) or vehicle control; 48 h later, we measured the levels of GLuc activity associated with virus-like-particles (VLPs) present in the CCS and in whole-cell lysates (WCLs) (Figure 3C). The Z budding efficiency (in %) was determined by the ratio of VLP-associated GLuc levels (Z_VLP_) and total GLuc levels (Z_VLP_ + Z_WCL_) times 100. BEZ-235, a known inhibitor of the Z budding activity, was used (10 µM) as a control [42]. DDD85646 had a strong inhibitory effect on LCMV and LASV Z-mediated budding.

We also examined whether DDD85646 exerted any virucidal activity on infectious LCMV virions. Treatment of infectious LCMV virions (10^5^ FFU) for 30 min at 37 °C with DD85646 at either 5 µM or 10 µM concentrations, which reduced the production of infections LCMV progeny by >5 logs (Figure 2B), did not significantly affect virion infectivity, whereas treatment with 0.5% NaOCl under the same conditions resulted in a complete loss of infectivity (Figure 3D).

### 3.4. Effect of DDD85646 on GP2-Mediated Fusion

**Figure 4:**
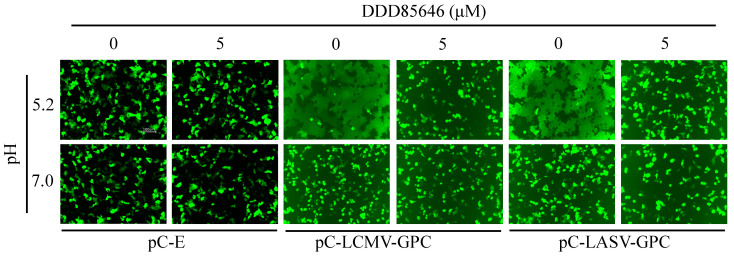
**Effect of DDD85646 on LCMV and LASV GP2-mediated fusion.** HEK293T cells were seeded in a M24-well plate at a density of 2.5 × 10^5^ cells/well. The next day, the cells were transfected with pC-LCMV-GPC, pC-LASV-GPC, or pC-E (1 μg/well), all conditions received pC-GFP (50 ng/well). At 5 h post-transfection, the cells were washed once with DMEM/10% FBS and treated with DDD85646 or VC. At 24 h post-transfection, the cell monolayers were treated with acidic (pH 5.0) or neutral (pH 7.2) medium for 15 min, then returned to neutral medium (DMEM/10% FBS) and examined for fusion over time. Once fusion was observed in the VC-treated samples, the cells were fixed for IF imaging with a 20X lens using the Keyence BZ-X710 imaging system.

Mammarenaviruses enter cells via receptor-mediated endocytosis [43,44,45]. In the acidic environment of the endosome, GP2 mediates a pH-dependent fusion event between viral and cellular membranes that results in the release of the vRNP into the cytoplasm of the cell, where virus replication and gene transcription take place. Myristoylation of SSP has been implicated in GP2-mediated fusion [14]; therefore, treatment with DDD85646 would be expected to interfere with GP2-mediated fusion. To examine this possibility, we transfected HEK293T cells with plasmids expressing LCMV (pC-LCMV-GPC) or LASV (pC-LASV-GPC) GPC or with an empty plasmid (pC-E) as a control, together with a plasmid expressing GFP (pC-GFP). At 5 h post-transfection, cells were treated with DDD85646 (5 µM) or vehicle control (VC), while the next day, the cells were exposed to acidic (pH 5), or neutral (pH 7.2) medium for 15 min and then returned to regular medium (pH 7.2), and cell fusion was monitored over time (Figure 4). Cells transfected with plasmids expressing LCMV or LASV GPC and exposed to pH 5 exhibited very strong fusion activity as determined by the syncytial formation revealed by the pattern of GFP expression. In contrast, treatment with DDD85646 resulted in abrogation of GP2-mediated fusion upon exposure to low pH.

### 3.5. Effect of DDD85646 on Z Protein Stability

**Figure 5:**
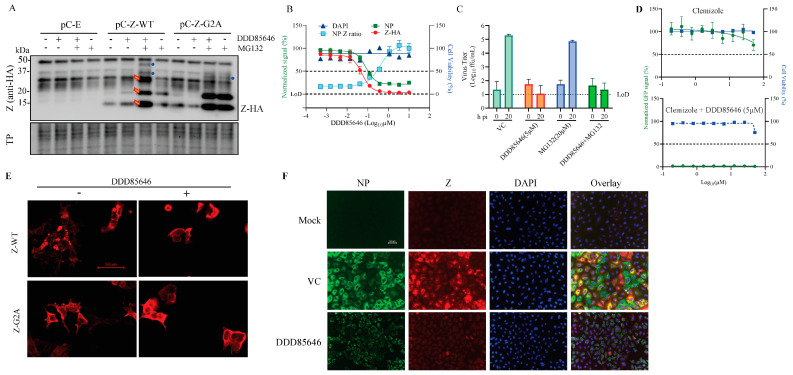
**Effect of DDD85646 on Z expression levels and subcellular distribution.** (**A**) HEK293T cells were seeded (4 × 10^5^ cells/M6-well) and transfected 16 h later with pCAGGS empty (pC-E), Z wild type (pC-Z-WT) or Z (G2A) (pC-Z-G2A) plasmids. At 24 h post-transfection, the cells were treated with DDD85646, MG132, or both, and at 24 h post-treatment, cell lysates were prepared and protein expression examined by Western blotting. Red arrows indicate Z protein oligomers; blue circles indicate Z protein-specific associated bands. (**B**) A549 cells were plated on 96-well clear-bottom plates (4.0 × 10^4^ cells/well). Sixteen hours later, the cells were infected with rLCMV/Z-HA (MOI 1), and serial dilutions (3-fold) of DDD85646 were added to cells, starting at 10 μM. At 72 h after the drug treatment, the cells were fixed with 4% PFA and stained with fluorescent antibodies for signal quantification; each point represents the mean of four biological replicates. (**C**) A549 cells were infected (MOI = 0.1) with rLCMV/GFP-P2A-NP. After a 90 min adsorption, the inoculum was removed, the cells were washed once, and medium containing either MG132 (20 µM), DD85646 (5 µM), or MG132 + DDD85646 was added to the cells. Cell culture supernatants were collected at 0 (after adsorption) and 24 h post-infection, and virus titers were determined by the FFA. (**D**) A549 cells were seeded at 4 × 10^4^ cells/well into a 96-well plate, infected with rLCMV/GFP-P2A-NP (MOI 0.05), and treated with incremental doses of clemizole or with clemizole in the presence of 5μM DDD85646. At 72 h pi, the cells were fixed, and the number of infected cells was determined by IF. (**E**) HEK293T cells were plated on poly-L-Lysine 96-well clear-bottom optically treated plates (2.0 × 10^4^ cells/well). The next day, the cells were transfected with LCMV Z-HA or the Z-G2A-HA mutant. At 24 h post-transfection (h pt), the media was replaced with DDD85646 (5 μM)-containing media, and at 48 h after drug treatment, the cells were fixed with 4% PFA. Images were taken using a Keyence BZX-710 microscope at 20× magnification, which were then zoomed in digitally to 1.5× magnification. The images were subjected to the Point–Spread Function (PSF) and then deconvolved using DeconvolutionLab2 (Fiji ImageJ, v154) with the following setting: Iterative Constraint Tikhonov–Miller algorithm at 100 iteration N, 1 step γ, and low reg. λ. (**F**) A549 cells were plated on 96-well clear-bottom optically treated plates (2.0 × 10^4^ cells/well). The next day, the cells were infected with rLCMV/Z-HA (MOI 0.05) and treated with DDD85646 (5 μM)-containing media 90 min later. At 72 h after drug treatment, the cells were fixed with 4% PFA and co-labeled with anti-HA (Z) and anti-NP antibodies and with DAPI for DNA; the Z protein is red, NP is green, and DNA is blue. The images have been zoomed in digitally to 3× magnification.

N-myristoylation of the Z protein is required for its budding activity. Therefore, we expected that cell-associated levels of Z protein would increase in DDD85646 compared with vehicle-treated cells, which would account for the reduced Z budding efficiency in the presence of DDD85646. To test this hypothesis, we transfected HEK 293T cells with plasmids expressing C-terminal HA-tagged versions of Z-WT or its mutant form, Z-G2A, that cannot undergo N-myristoylation. Intracellular levels of Z-WT, but not of Z-G2A, were slightly increased in the presence of DDD85646 (Figure 5A), which was difficult to reconcile with the corresponding strong reduction in Z budding efficiency. This led us to examine whether Z protein stability was compromised in the presence of DDD85646. We found that cell-associated expression levels of Z-WT were significantly increased upon treatment with the 20S proteasome inhibitor MG132 (20 μM) in the presence of DDD85646 (5 µM), supporting the hypothesis that, as with other N-myristoylated proteins, Z expression levels is subjected to the control of the glycine-specific N-degron pathway involved in the quality control of protein N-myristoylation [16]. Consistent with this view, expression levels of Z-G2A, which does not undergo N-myristoylation, were similarly increased in the absence and presence of DDD85646 following treatment with MG132. We observed that the inhibition of the proteasome pathway by MG132 treatment resulted in increased levels of monomer and dimer forms of both the WT and G2A forms of Z but only of trimers of Z-WT (Figure 5A). Further studies will examine the implications of this observation; however, this is outside the scope of the present work.

To further investigate the role of myristoylation on Z expression, we examined the effect of DDD85646 on Z expression levels in LCMV-infected cells. Because of the lack of suitable Z-specific antibodies, for this experiment, we used a rLCMV plasmid expressing a C-terminal HA-tagged Z (rLCMV/Z-HA). We infected A549 cells with rLCMV/Z-HA (MOI 1) and treated them with the indicated DDD85646 concentrations; at 72 h pi, we determined the NP:Z ratios by measuring the immunofluorescent signals obtained with a mouse monoclonal antibody to HA (Z protein) and the rat monoclonal antibody VL4 to LCMV NP. Consistent with the inhibitory effect of DDD85646 on LCMV multiplication, expression levels of both Z and NP were reduced by DDD85646 treatment in a dose-dependent manner. However, the normalized NP:Z ratio was increased by DDD85646 treatment in a dose-dependent manner, supporting that Z, but not NP, was targeted for degradation upon DDD85646-mediated inhibition of myristoylation (Figure 5B). To assess whether proteasome-mediated degradation of Z protein contributed to the antiviral activity of DDD85646, we compared the effect of MG132, in the presence and absence of DD85646, on the production of infectious LCMV progeny. Treatment with MG132 did not significantly affect the inhibitory effect of DDD85646 on the production of infectious LCMV progeny (Figure 5C), and consistent with previous findings [46], MG132 did not significantly affect the production of infectious LCMV progeny.

Heme oxygenase 2 (HO-2) has been shown to negatively regulate the functions of myristoylated proteins by influencing the localization and trafficking of its binding partners as well as targeting them for degradation [47,48]. Thus, pharmacological inhibition of the interaction of HO-2 with HIV-1 MA protein was shown to result in the enhanced production of HIV-1 virions [47]. We therefore examined the effect of the validated HO-2 inhibitor clemizole [49,50,51,52] on LCMV multiplication using rLCMV/GFP-P2A-NP (MOI 0.05). We observed that a high (50 µM) concentration of clemizole had a rather modest effect on LCMV multiplication at 72 h pi, whereas the inhibitory effect of DDD85646 (5 µM) on LCMV multiplication was not affected in the presence of clemizole (Figure 5D).

### 3.6. Effect of DDD85646 on the Subcellular Distribution of Z Protein

We next assessed the effect of DDD85646 treatment on the subcellular location of the Z protein. We first examined the effect of DDD85646 in HEK293T cells transfected with the pCAGGS plasmid expressing C-terminal HA-tagged versions of WT (Z-WT) or mutant G2A (Z-G2A) Z proteins. At 48 h post-treatment, the cells were fixed and stained with an HA antibody. We observed that the Z protein exhibited a punctate appearance in the absence of DDD85646, while its distribution was homogeneous and lacked puncta in the presence of DDD85646 (5 µM). This latter distribution was also observed in cells transfected with Z-G2A and was not affected by DDD85646 treatment (Figure 5E), a finding consistent with published data on the subcellular distribution of LASV Z-WT and Z-G2A [53]. Next, we examined the effect of DDD85646 on the subcellular localization of Z and NP in LCMV-infected cells. A549 cells were infected with rLCMV/Z-HA (MOI 0.05) and treated with DDD85646 (5 μM) or vehicle control. Mock-infected cells served as an additional control. At 48 h pi, cells were fixed and co-labeled with anti-HA (Z) and anti-NP antibodies. Consistent with the results of studies examining the NP:Z ratio (Figure 5B), both NP and Z expression levels were reduced upon treatment with DDD85646, but Z exhibited a much higher degree of inhibition (Figure 5F), further supporting the idea that inhibition of N-myristoylation targets Z for degradation. In cells treated with DDD85646 but not with the vehicle control, NP exhibited a punctate distribution (Figure 5F), a finding that might reflect that myristoylated Z or host cell proteins can influence the NP subcellular distribution [30,54,55,56].

### 3.7. Contribution of the Type 1 Interferon (T1IFN) Response to DDD85646-Mediated Restricted LCMV Multiplication

**Figure 6:**
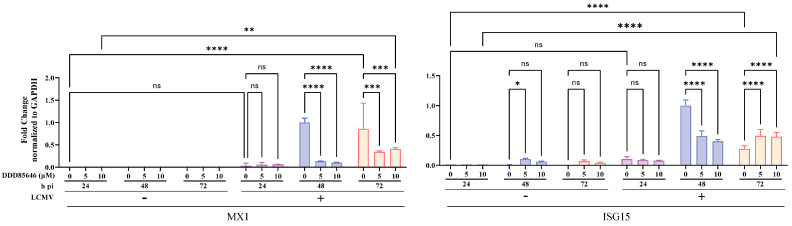
Effect of DDD85646 on the expression of ISGs. A549 cells were seeded at 2 × 10^5^ cells/well in an M12-well plate, infected with LCMV/GFP-P2A-NP (MOI 0.05), and treated with DDD85646 (5 or 10 µM) or with VC. At the indicated time points, total cellular RNA was isolated and analyzed by RT-qPCR (10 ng/sample; three technical replicates). The RT step was performed using random hexamers, followed by quantitative PCR with specific primers for ISGs MX1, or ISG15 and the cell housekeeping gene GAPDH. MX1 and ISG15 fold changes were normalized to GAPDH. The repeated-measures analysis of variance with mixed-effect analysis and Dunnet’s correction for multiple comparisons were used. Statistically significant values: ns *p* > 0.05, * *p* ≤ 0.05, ** *p* < 0.01, *** *p* < 0.001, **** *p* < 0.0001.

N-myristoylation has been implicated in host innate immunity defense mechanisms against microbial and viral infections [57,58]. As with many other viruses, LCMV encodes gene products that are potent viral counteracting factors of the host cell type I interferon (T1IFN) response [34,59]. We, therefore, examined whether DDD85646 induced innate immune responses that could not be counteracted by LCMV interferon-counteracting factors. We used RT-qPCR to quantify the levels of MX1 and ISG15 transcripts in response to DDD85646 treatment in mock- and LCMV-infected cells. Treatment with concentrations (5 and 10 µM) of DDD85646 that caused a potent inhibition of LCMV multiplication did not affect the levels of MX1 or ISG15 in mock-infected cells (Figure 6). A modest (≤1.5 fold), but significant, upregulation of MX1 and ISG15 mRNAs was observed in LCMV-infected cells treated with DDD85646, which likely reflects reduced levels of production of the anti-T1IFN viral factor NP [34,59].

### 3.8. Contribution of NMT1 and NMT2 Isozymes to LCMV Multiplication

**Figure 7:**
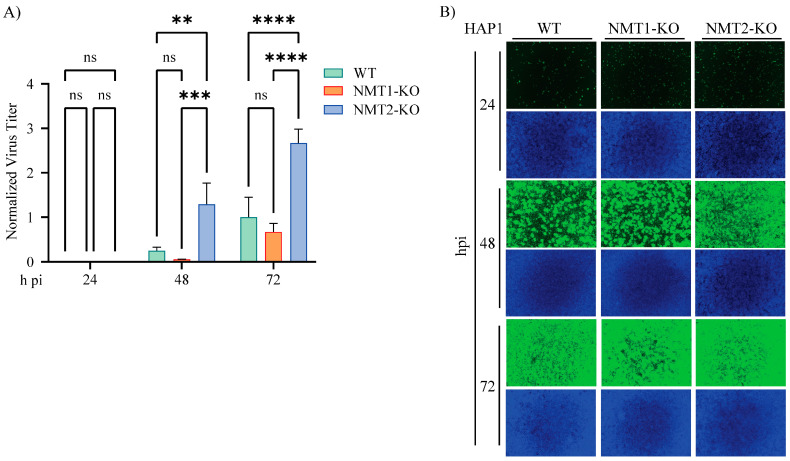
**Contribution of NMT1 and NMT2 isozymes to LCMV multiplication**. HAP1 NMT WT, NMT-1-KO, or NMT-2-KO cells were seeded at 2 × 10^5^ cells/well in an M12-well plate and infected with LCMV/GFP-P2A-NP (MOI 0.05). At the indicated time points, CCS was collected for virus titration. Virus titers were normalized to titers at 72 h pi in the VC-treated sample, which was assigned the value of 1 (**A**), and the cells were fixed for immunofluorescence imaging (**B**). The repeated-measures analysis of variance with mixed-effect analysis and Tukey’s correction for multiple comparisons were used. Statistically significant values: ns *p* > 0.05, * *p* ≤ 0.05, ** *p* < 0.01, *** *p* < 0.001, **** *p* < 0.0001.

To investigate a possible differential role of the two ubiquitously expressed N-myristoyltransferase isozymes NMT1 and NMT2 on LCMV multiplication, we assessed the multiplication of LCMV in the near-haploid fibroblast human cell line HAP1, in which the expression of either NMT1 or NMT2 has been abrogated via CRISPR/Cas9-induced knockout (KO) lines [12]. Lack of NMT1 or NMT2 had a similar modest effect on the production of infectious LCMV progeny (Figure 7A) and cell propagation (Figure 7B) in a multi-step growth kinetics assay. However, we observed a slight, but significant, differential increment at 48 h pi in NMT2-KO cells, suggesting that NMT1 might exert a dominant effect on the myristoylation of LCMV proteins required for normal levels of virus multiplication, a finding similar to that reported for HIV [60] and CVB3 [12].

### 3.9. Effect of DDD85646 on the Multiplication of Other Mammarenaviruses

**Figure 8:**
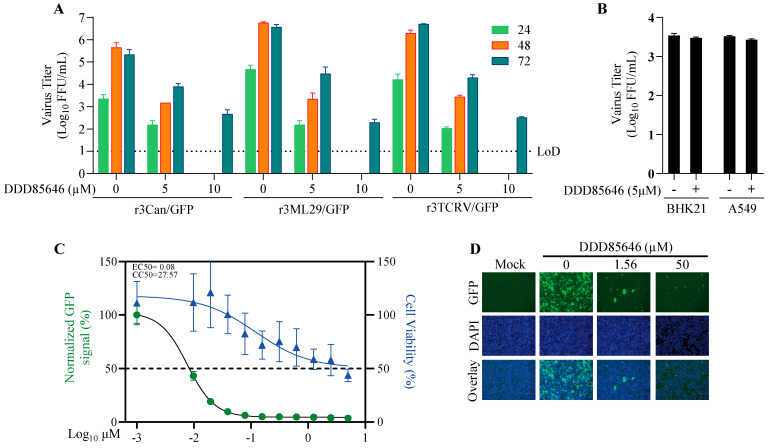
Effect of DDD85646 on the multiplication of the mammarenaviruses JUNV, TCRV, ML29, and LASV, and the flavivirus ZIKV. (**A**) A549 cells were infected with the indicated mammarenavirus and treated with DDD85646 (5 or 10 µM) or VC. At the indicated h pi, titers of infectious virus in the cell culture supernatants were determined by the FFA. (**B**) A549 and BHK21 cells were seeded into M24-well plates at a density of 1 × 10^5^ cells/well and infected with ZIKV (MOI 0.1) for 1 h, then treated with DDD85646 (5 µM). At 24 h pi, supernatants were collected, and virus titers were determined by the FFA. Titers of technical duplicates were log-transformed and plotted as the mean ± SD (error bars). (**C**) A549 cells were seeded at 4 × 10^4^ cells/well into a 96-well plate, infected with r3LASV/GFP (MOI 0.05), and treated with DDD85646 at the indicated concentrations. At 72 h pi, the cells were fixed, and the numbers of infected cells were determined by immunofluorescence. Numbers of infected cells were normalized to those of vehicle control (VC)-infected cells and expressed as a % of infected cells (viral infection). Cell viability was estimated based on the signal of DAPI staining. Results show the percentage of infected cells (four biological replicates). EC50 and CC50 values were calculated using a variable slope (based on four parameters) model (Prism10). Results show the mean and SD of four replicates. Representative immunofluorescence images of the dose-response assay of selected doses of DDD85646 are shown (**D**).

Related viruses are likely to rely on the same host cell factors for their activities; therefore, compounds targeting these host cell factors provide an opportunity for the development of broad-spectrum antiviral therapeutics. To assess whether NMT inhibitors exhibit antiviral activity against other mammarenaviruses, we examined the effect of DDD85646 on the multiplication of the live-attenuated vaccine strain (Candid#1) of JUNV, the LF live-attenuated vaccine candidate reassortant ML29 carrying the L segment from the non-pathogenic Mopeia virus and the S segment from LASV, and the Tacaribe virus (TCRV). For these experiments, we used tri-segmented versions of Candid#1 (r3Can), ML29 (r3ML29), and TCRV (r3TCRV) expressing the GFP reporter gene. DDD85646 exhibited a potent dose-dependent inhibitory effect against these three mammarenaviruses (Figure 8A). In contrast, and consistent with previous findings [61], Zika virus (ZIKV) multiplication in BHK21 and A549 cells was not affected by treatment with DDD85646 (Figure 8B). DDD85646 was also effective against highly pathogenic mammarenaviruses and potently inhibited multiplication in A549 cells of the HF-causing mammarenavirus LASV (Figure 8C,D).

## 4. Discussion

Myristoylation of mammarenavirus SSP and Z protein plays a critical role in GP2-mediated fusion and Z-mediated budding, respectively, two processes required for the completion of the mammarenavirus life cycle [1]. Myristoylation of mammalian proteins is mediated by N-myristoyltransferases (NMT) 1 and 2 [12]. Here we have presented evidence that DDD85646 [12,13], a validated specific pan-NMT inhibitor, exhibits a potent dose-dependent inhibitory effect on the multiplication of LCMV as well as of other mammarenaviruses, including the HF-causing LASV and JUNV. We also observed potent anti-LCMV and anti-LASV activity with IMP-1088, a different specific pan-NMT inhibitor [11,62] (Figure S1). DDD85646-mediated inhibition of Z and SSP myristoylation results in impaired viral assembly and budding as well as the GP-mediated fusion event required for the completion of the virus cell entry process, which is reflected in the reduced virus propagation following infection at an MOI of 0.05 (≤5% cells infected in the first round of infection). Accordingly, treatment with DDD85646 resulted in reduced levels of virus replication and transcription at all the times examined, starting at 24 h pi. DDD85646 did not affect virus cell entry and had a modest impact (20% reduction) on viral RNA synthesis mediated by vRNP in the LCMV cell-based minigenome system, whereas Z-mediated budding and GP2-mediated pH-dependent fusion were strongly inhibited by DDD85646.

Myristoylation of Z is required for its budding activity; therefore, treatment with DDD85646 would be expected to result in increased levels of cell-associated Z protein. Accordingly, we observed an increase, though modest, of cell-associated Z protein following treatment with DDD85646. Levels of cell-associated Z protein in cells treated with DDD85646 markedly increased upon treatment with the proteasome inhibitor MG132, supporting the hypothesis that non-myristoylated Z protein is targeted for degradation via the proteasome. The E3 ubiquitin ligase ITCH has been shown to interact with LASV and LCMV Z proteins and contribute to virus assembly and budding [63]. Thus, it is plausible that ITCH interaction with non-myristoylated Z, but not with myristoylated Z, promotes the proteasome-mediated rapid degradation of Z; however, this prediction warrants further investigation, which is outside the scope of the current work. Preferential degradation of Z in the presence of DDD85646 was also reflected by the increased NP:Z ratios, determined by immunofluorescence, in response to treatment with increased concentrations of DDD85646. This experiment was performed using a C-terminal HA-tagged version of the Z protein. However, it is highly unlikely that the HA tag influenced the outcome of the experiment, since growth kinetics and peak titers of rLCMV-Z-HA were not significantly affected. Bioinformatic analysis revealed that both HA-tagged Z-WT and Z-G2A mutant proteins have five putative degrons [64]. The functional degron region, which includes parts of the tripartite model and the tertiary structure necessary for E3 ligase engagement, is predicted to be located within 40 amino acids of the degron motif, whereas the HA tag was positioned more than 50 amino acids away from lysine 38 (K38) of the degron motif, supporting the exclusion of HA as an interfering factor. Moreover, experimental evidence supports the assumption that the HA tag does not interfere with C-terminal degrons in other proteins across different systems of eukaryotes [65,66,67,68], and we have shown that an LCMV Z protein with a C-terminal HA tag is fully functional in cell-based assays virus [69]. Computational analysis of destabilizing N-terminal motifs indicated that, in contrast to glycine, an alanine at position 2 does not substantially contribute to protein destabilization [16]. However, the Z protein containing the G2A mutation remained sensitive to MG132-mediated degradation, an intriguing finding that warrants studies beyond the scope of the present work.

Myristoylation of the SSP is required for GP2-mediated fusion, which could account for the inhibitory effect of DDD85646 on GP2-mediated fusion. However, we cannot rule out that proteasome-mediated degradation of SSP also contributed to the restricted GP2-mediated fusion in the presence of DDD85646.

The activity of myristoylated proteins can be negatively regulated by HO-2 [47]. However, the anti-LCMV activity of DDD85646 was not affected by clemizole, a validated inhibitor of HO-2 [49,50,51,52]. DDD85646 did not trigger ISGs, supporting the T1IFN-independent antiviral activity of DDD85646. Comparison of LCMV multiplication between WT, NMT1-KO, and NMT2-KO HAP1 cells indicated that NMT1 might play a dominant role in the myristoylation of LCMV proteins that is required for normal levels of virus multiplication. It is possible that in the absence of NMT-2, the expression of NMT1 is substantially upregulated in HAP1 cells, which could account for the more efficient production of LMCV in these cells.

Results from studies of the role of NMT in cancer cell lines support a large efficacy window between host and virus due to substantial differences in the rate of protein turnover [70]. Upon initiation of treatment with an NMT inhibitor, pre-existing N-myristoylated host proteins must be degraded before the inhibitor can have an impact on the cell physiology, a process that could take several days. It should be noted that the concept of targeting a core host-lipidation process to block viral replication has been validated in a phase 2A trial of an inhibitor of host prenylation of the hepatitis D virus [71]. DDD85646 exhibited a similarly potent antiviral activity against other tested mammarenaviruses, including the highly pathogenic LASV, indicating its potential pan-mammarenavirus antiviral activity. The observation that mammarenaviruses have evolved to depend on host myristoylation to complete some of the steps of their life cycle suggests that this mode of action might circumvent the development of resistance to a drug that targets NMT because viral mutations would not influence an inhibitor’s potency against a host enzyme. Notably, a recent human phase I trial has shown that the oral, highly bioavailable small-molecule NMT inhibitor PCLX-001 is safe and well tolerated at concentrations that result in PCLX-001 plasma concentrations over its EC_90_ [28]. Progress on NMT inhibitor-based cancer therapies can facilitate the repurposing of NMT inhibitors as antiviral agents against human pathogenic mammarenaviruses. In studies beyond the scope of the present work, we will examine the antiviral effect of PCLX-001 in a mouse model of LCMV infection.

## Data Availability

Data are contained within the article and supplementary materials.

## Supplementary Materials

The following supporting information can be downloaded at: https://www.mdpi.com/article/10.3390/v16091362/s1, Figure S1. Dose-dependent effect of the NMT inhibitor IMP1088 on LCMV and LASV multiplication in A549 cells. A549 cells were seeded at 4 × 10^4^ cells/well into a 96-well plate, infected with rLCMV/GFP-P2A-NP (MOI 0.05), and treated with serial dilutions (2-fold) of IMP-1088. At 72 h pi, the cells were fixed, and the numbers of infected cells were determined by IF. Numbers (%) of infected cells were normalized to vehicle control (VC)-infected cells. Total cell numbers were determined by the MTS/formazan bioreduction assay using the Cytation 5 reader. Results show the percentage of infected cells (four biological replicates). EC50 and CC50 were calculated using a variable slope (based on four parameters) model (Prism10).
