# Supplementary figures and images for: Cellular N-Myristoyl Transferases Are Required for Mammarenavirus Multiplication
Source: Viruses. 2024 Aug 26;16(9):1362. doi: 10.3390/v16091362 (PMC11436053; doi:10.3390/v16091362)

Figure S1. Dose-dependent effect of NMT inhibitor IMP1088 on LCMV and LASV multiplication in A549 cells

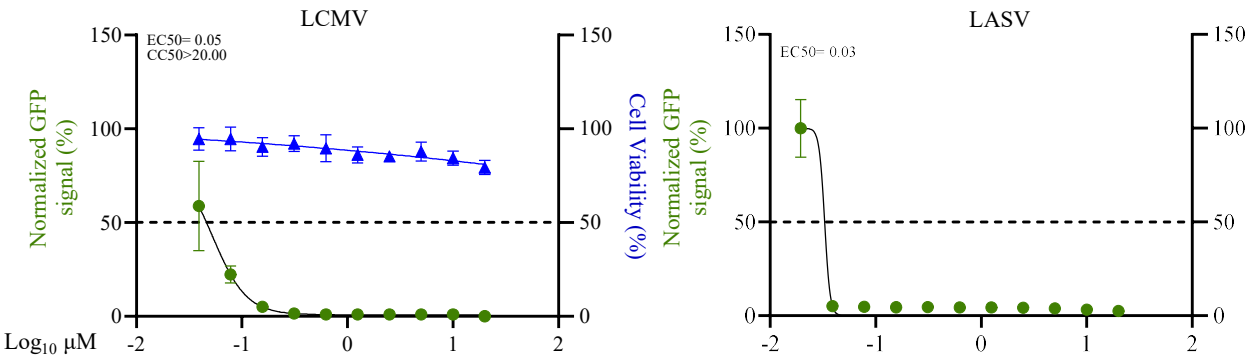

Supplement: Supplementary file 1 [file viruses-16-01362-s001.zip › viruses-3165476-supplementary.pdf]
